# Different biological activity of CD154-SIVgp41 fusion protein vaccine component in naïve or pre-immune individuals

**DOI:** 10.1186/1742-4690-9-S2-P339

**Published:** 2012-09-13

**Authors:** LM Smith, LM Parodi, VL Hodora, LD Giavedoni

**Affiliations:** 1 Texas Biomedical Research Institute, San Antonio, TX, USA

## Background

Trimeric gp41 might be an important target for neutralizing antibodies; however, the poor immunogenicity of this region may be due to its lack of exposure on native virus. CD154 (CD40L) is a trimeric glycoprotein found on activated CD4 T-cells that binds to CD40 on APCs and leads to B-cell activation and differentiation to plasma cells. We designed a novel immunogen with the potential for inducing neutralizing antibodies to gp41 and for stimulating activity on APC and B-cells.

## Methods

A flexible (FL) and helical (HL) linkers were used to join CD154 and SIV gp41, which are trimeric glycoproteins with opposing polarities. Recombinant vaccinia viruses (VVs) were engineered to express CD154FLgp41 and CD154HLgp41 glycoproteins. PBMCs from SIV naïve and immune macaques were exposed to wild type VV (VVwt) or to recombinant VVs expressing SIV Gag, gp160, and Nef (VVgen), or SIV Gag and Nef, and CD154-gp41 fusion proteins. Cytokine production and cell activation were analyzed by Luminex and flow cytometry, respectively.

## Results

Both linkers allowed proper protein folding and CD154 biological activity. Compared to VVwt, VVgen, and VVCD154FLgp41, PBMCs from naïve macaques exposed to VVCD154HLgp41 expressed higher levels of IL-6, IL-1Rα, IL-1β, RANTES, GRO-α, TNF-α, IL-8, IP-10 and IL-10. In contrast, PBMCs from SIV-immune macaques expressed more IL-6, MIP-1α, MIP-1β, INF-α, IL-1β, and MCP-1. Interestingly, lymphocytes from SIV-immune macaques were activated by VVCD154HLgp41 and VVCD154HLgp41 more than cells exposed to SIVgen.

## Conclusion

Fusion proteins of CD154 and gp41 with either HL or FL assembled into trimers and activated immune cells. However, there was a differential cytokine expression for the fusion protein containing the HL, including chemokines that inhibit HIV entry. This increased biological activity may indicate that the helical linker allows for a more functional folding of the CD154 moiety. The CD154HLgp41 fusion protein will be tested in NHP as a novel vaccine component.

